# Supplementary material for: Causal Relationship between Aging and Anorexia Nervosa: A White-Matter-Microstructure-Mediated Mendelian Randomization Analysis
Source: Biomedicines. 2024 Aug 16;12(8):1874. doi: 10.3390/biomedicines12081874 (PMC11351342; doi:10.3390/biomedicines12081874)
Supplement: Supplementary file 1 [file biomedicines-12-01874-s001.zip › Supplementary Figure/Figure S8/Leaveoneout plot of MR analysis for TL to ubm-b-1664.pdf]

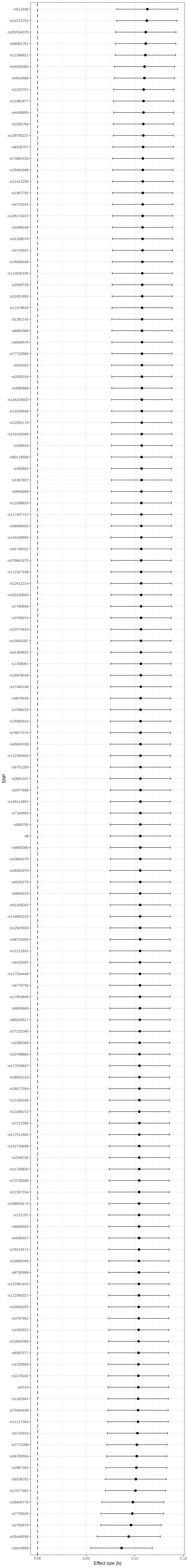
rs611646
rs10112752
rs202034370
rs56061761
rs11584821
rs34550383
rs4616688
rs1023767
rs11991877
rs4498805
rs2282764
rs139795227
rs8105767
rs73581419
rs78491606
rs11412296
rs1907702
rs4731541
rs185174247
rs9398196
rs41269079
rs4743037
rs76666449
rs113525195
rs2056726
rs12451892
rs11579626
rs1291143
rs6881568
rs6584579
rs77732866
rs932002
rs2555104
rs3093888
rs144204502
rs13230646
rs12932179
rs142426306
rs939916
rs80116508
rs450962
rs1957937
rs9940099
rs11699829
rs117407747
rs59409453
rs143190905
rs61748181
rs376641875
rs111527438
rs12412214
rs150150565
rs7790856
rs3785074
rs10774624
rs10845387
rs41304832
rs7209057
rs16978028
rs17445108
rs9878436
rs7099229
rs76065543
rs79977579
rs45604339
rs112394943
rs6751209
rs3891167
rs2977608
rs145114957
rs7164950
rs869785
All
rs9955360
rs10840270
rs28363070
rs4530278
rs9600019
rs61405042
rs116863223
rs12925933
rs66731853
rs11212631
rs8102497
rs117034449
rs6776756
rs17803849
rs6659669
rs80324517
rs77231040
rs1985369
rs10768683
rs117630647
rs28502153
rs28577594
rs11426156
rs11085072
rs7221585
rs117512405
rs142730696
rs2538745
rs11769630
rs73730598
rs11557154
rs188918174
rs131797
rs6669563
rs4695407
rs76219171
rs10805346
rs6790988
rs137901416
rs111950327
rs10905255
rs3767952
rs1003322
rs13062095
rs6587577
rs2230590
rs2276182
rs4724
rs1332941
rs75664430
rs11117354
rs5742915
rs7772289
rs56799554
rs2967355
rs6538702
rs17677991
rs35640778
rs7705526
rs2763979
rs35446936
rs9419958
SNP
0.00
0.05
0.10
0.15
Effect size (b)
